# Morphology and Quantitative Monitoring of Gene Expression Patterns during Floral Induction and Early Flower Development in *Dendrocalamus latiflorus*

**DOI:** 10.3390/ijms150712074

**Published:** 2014-07-07

**Authors:** Xiaoyan Wang, Xuemei Zhang, Lei Zhao, Zhenhua Guo

**Affiliations:** 1 China Southwest Germplasm Bank of Wild Species, the Key Laboratory of Biodiversity and Biogeography, Kunming Institute of Botany, Chinese Academy of Sciences, Kunming 650201, China; E-Mails: wangxiaoyan@mail.kib.ac.cn (X.W.); zhangxuem@mail.kib.ac.cn (X.Z.); zhaolei@mail.kib.ac.cn (L.Z.); 2 Kunming College of Life Science, University of Chinese Academy of Sciences, Kunming 650201, China

**Keywords:** *Dendrocalamus latiflorus*, floral induction, early floral development, morphological characteristics, gene expression profiling, molecular marker

## Abstract

The mechanism of floral transition in bamboo remains unclear. *Dendrocalamus latiflorus* (Bambusease, Bambusoideae, Poaceae) is an economically and ecologically important clumping bamboo in tropical and subtropical areas. We evaluated morphological characteristics and gene expression profiling to study floral induction and early flower development in *D*. *latiflorus*. The detailed morphological studies on vegetative buds and floral organography were completed using paraffin sectioning and scanning electron microscopy. The 3 mm floral buds commence the development of stamen primordia and pistil primordium. Furthermore, homologs of floral transition-related genes, including *AP1*, *TFL1*, *RFL*, *PpMADS1*, *PpMADS2*, *SPL9*, *FT*, *ID1*, *FCA*, and *EMF2*, were detected and quantified by reverse transcriptase PCR and real-time PCR in vegetative and floral buds, respectively. Distinct expression profiles of ten putative floral initiation homologues that corresponded to the developmental stages defined by bud length were obtained and genes were characterized. Six of the genes (including *DlTFL1*, *DlRFL*, *DlMADS2*, *DlID1*, *DlFCA*, *DlEMF2*) showed statistically significant changes in expression during floral transition. *DlAP1* demonstrated a sustained downward trend and could serve as a good molecular marker during floral transition in *D. latiflorus*. The combined analysis provided key candidate markers to track the transition from the vegetative to reproductive phase.

## 1. Introduction

Bamboo is a member of the perennial evergreens in the grass family (Poaceae) and one of the most important forest resources [1]. There are approximately 1400 species and 88 genera of bamboo worldwide, of which 534 species and 34 genera have been reported in China [2]. Bamboo plants possess certain unique biological characteristics which significantly differ from other land plants, including fast shoot growth, a long duration of vegetative phase (3 to 120 years depending on the species), semelparity, and mast flowering [3,4]. These traits exacerbate the problem of wild bamboo sustainability and threaten the subsistence of animals and human communities that depend on these plants [5]. *Dendrocalamus latiflorus* (Bambusease, Bambusoideae, Poaceae), an arborescent and perennial grass with sporadic flowering, is the most widely distributed and cultivated grass in southern China. It is a representative species of clumping bamboo [6]. The shoots, which are rich in nutrition, are traditional vegetables in southern China.

Floral patterning and the specification of floral organs are key processes during plant life. When plants initiate flowering, the shoot apical meristem (SAM) is transformed into an inflorescence meristem (IM). The IM produces the floral meristems (FMs), which give rise to floral organs [7]. In grasses, the SAM to IM transition is characterized by clear changes in developmental and morphological traits [8]. Processes of inflorescence and spikelet development in grasses differ considerably from those in other monocotyledonous and dicotyledonous species [9]. The structural units of the grass ﬂower are spikelets and ﬂorets, which are different from those of eudicots. The primary unit of the grass inﬂorescence consists of spikelets, which are comprised of glumes (bract-like organs) and ﬂorets. The ﬂoret consists of a lemma, palea, lodicules, stamens, and a carpel. Both lemma and palea are grass-speciﬁc organs, but their identities are still controversial [7,10,11]. Several studies have reported the expression of ﬂoral meristem identity genes during ﬂower development [12,13,14]. However, in contrast to a large amount of information concerning the molecular mechanism of floral transition and flower development in several other woody plants, very limited molecular studies have been undertaken on floral transition and early flower development of bamboo, like *D. latiflorus*.

Using *Arabidopsis thaliana* as a eudicot model, molecular and genetic analyses revealed that regulation of floral transition largely depends on environmental cues as well as endogenous factors [15]. The transcriptional regulation of several genes such as the FM identity gene *LEAFY* and the “flowering-time” genes *FLOWERING LOCUS T* (*FT*) and *APETALA1* (*AP1*) determined the floral fate of nascent lateral primordia produced by the SAM, followed by ﬂoral morphogenesis [16]. *FLORICAULA*/*LFAFY*-like (*RFL*) gene is the homology of *LEAFY* in rice [17]. The FT protein moves from leaves to the shoot apex, where it interacts with the transcription factor encoded by *FLOWERING LOCUS D* (*FD*) to activate genes that determine floral organ identity and consequently induces flowering [18,19,20]. The activity of the IM identity gene *TERMINAL FLOWER1* (*TFL1*) can restrain the expression of *AP1* and *LFY*, and therefore the formation of a terminal flower. Analysis of transgenic ryegrass overexpressing *LpTFL1* showed that *LpTFL*-mediated control of floral transition [21]. Page *et al.* (1999) supported a model in which flowering time control protein FCA (*FCA*) function promotes ﬂowering in multiple pathways, one of which leads to activation of *LFY* and *AP1*, while the other acts in parallel with *LFY* and *AP1* [22]. Xu *et al.* (2010) cloned a novel *EMBRYONGIC FLOWER2* gene, *DlEMF2*, which was isolated from *D. latiﬂorus* and transformed to transgenic *Arabidopsis* plants, resulting in early-ﬂowering phenotypes [23]. According to Bian’s study, both MIKCC MADS-domain protein PpMADS1 (*PpMADS1*) and MIKCC MADS-domain protein PpMADS2 (*PpMADS2*) are involved in floral transition in *Phyllostachys praecox* [1]. The INDETERMINATE protein (ID1) plays a key role in regulating the transition to flowering in maize [24]. Single and double mutant phenotype analysis showed that *Squamosal promoter-binding-like protein 9* (*SPL9*) gene acts redundantly in controlling the juvenile-to-adult phase transition [25].

Nevertheless, the morphological structures of the vegetative and floral buds in *D. latiflorus* have not yet been well identified. The juvenile-to-adult phase transition is gradual and rather subtle, but can generally be followed by several morphological traits and molecular markers. During observation of biological characteristics of flowering ramets and anatomy of *D. latiflorus*, differences between the vegetative and reproductive buds can be distinguished. In the present study, quantitative reverse transcriptase PCR (qRT-PCR) was used to determine the expression levels of *TFL1*, *RFL*, *AP1*, *FCA*, *FT*, *ID1*, *SPL9*, *PplMADS1*, *PpMADS2*, and *EMF2* homologues in *D. latiflorus* during ﬂoral transition and flower formation. In addition, we conducted sequence and phylogenetic analysis of *TFL1*, *RFL*, and *AP1*-like in *D. latiflorus*. A series of landmark events in the development of morphological characters and the changes in gene expression were found during floral induction and early floral development. Together, these can be used to predict the identity of the buds that gave rise to vegetative or reproductive state.

## 2. Results and Discussion

### 2.1. Morphological Studies of the Transition from the Vegetative Bud to Inflorescence Initiation and Flower Ontogeny

The studies of morphological development of vegetative and floral buds in *D. latiflorus* are based on the methods of paraffin sectioning and scanning electron microscopy (SEM) analysis [26]. We classified floral induction and early floral development into five stages. All vegetative buds were placed in stage 1 and floral buds <5 mm were divided into stages 2–5: flower buds 0–2 mm length were categorized in stage 2, buds 2–3 mm in stage 3, buds 3–4 mm in stage 4, and buds 4–5 mm in stage 5.

**Figure 1:**
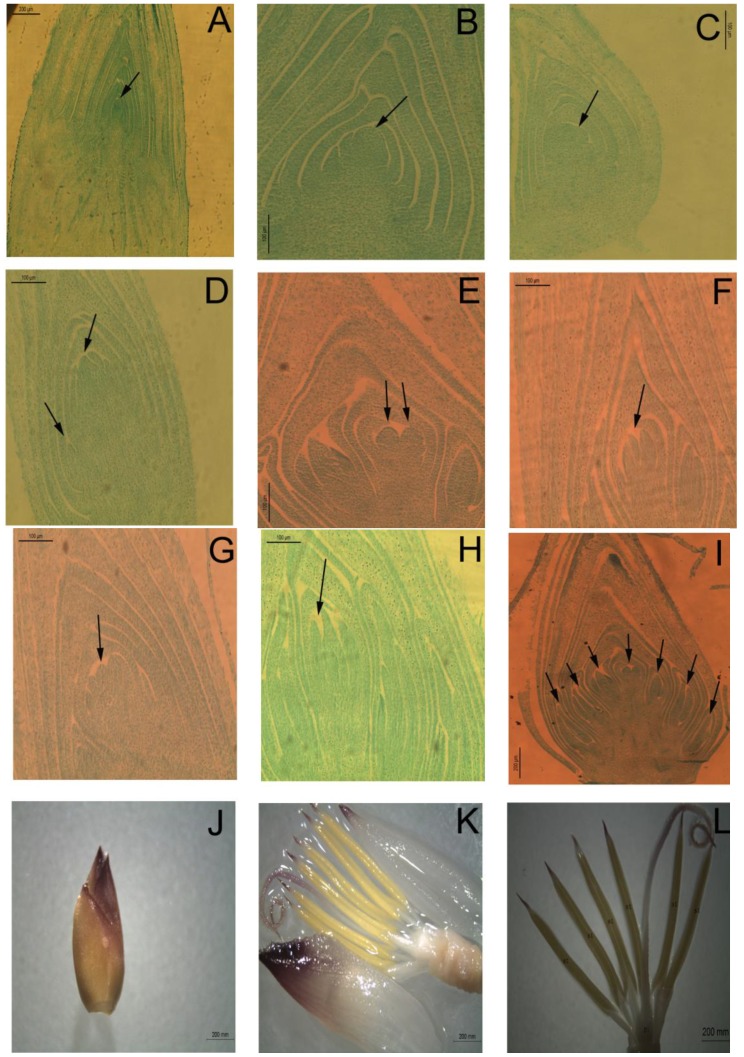
Morphogenesis of vegetative buds and floral buds of *Dendrocalamus latiflorus* according to the paraffin section inspection: (**A**,**B**) Vegetative buds; vegetative shoot apex in meristem (arrowhead); (**C**–**L**) Floral buds. (**C**) Initial stage of inflorescence meristem. Inflorescence meristem is wider than the vegetative shoot apex in meristem (arrowhead); (**D**) Lemma and palea primordial formation (arrowhead); (**E**,**F**) Carpel primordium and ovule (arrowhead) formation; (**G**) Initiation of stamen primordium (arrowhead); (**H**) Primordia of stamens and pistil continuing to develop and rapidly increasing in size (arrowhead); (**I**) A spikelet with florets (arrowhead); (**J**) External morphological characteristics of spikelets; (**K**) External morphological characteristics of floret (six stamens, a stigma, a lemma and a palea); (**L**) Six stamens and a pistil with feathery stigma. (**B**–**H**): The bar displayed 100 μm; (**A**,**I**,**J**–**L**): The bar displayed 200 μm

**Figure 2:**
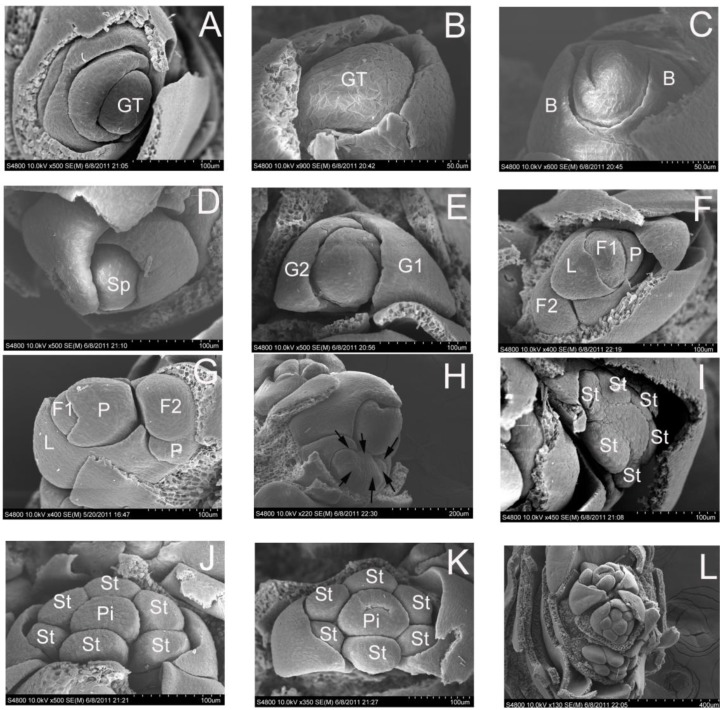
Morphogenesis of vegetative buds and spikelet and florets of *D. latiflorus* based on scanning electron microscopy (SEM) observations. (**A**,**B**) The apical growing tip on vegetative buds (GT-growth tip); (**C**) Bract primordium beginning differentiation (B-bract); (**D**) Initiation of spikelet primordium (Sp, spikelet primordium); (**E**) Initiation of glume primordium (G1, primordium of glume 1; G2, primordium of glume 2); (**F**,**G**) Initiation of lemma primordium of floret 1 (L, lemma; P, palea; F1, floret 1; F2, floret 2); (**H**,**I**) Initiation of stamen primordia (arrowhead) (St, stamen primordium); (**J**,**K**) Pistil and stamen primordia continuing developing and differentiating (St, stamen primordium; Pi, pistil). (**L**) A spikelet with some florets; a floret with six stamens and one pistil.

Stage 1 was characterized by a vegetative meristem in which there is a growth tip in the central region (Figure 1A,B and Figure 2A,B). During the transition from vegetative to reproductive growth, the SAM is converted to IM. At stage 2, the meristem was dome shaped and gave rise to spikelet primordium (Figure 1C). The spikelet meristem differs from the vegetative meristem in that the spikelet meristem (Figure 1C) is taller and a little wider than the vegetative meristem (Figure 1A,B). The initiation of bract primordium occurred in the phase (Figure 1D and Figure 2C). Each of these primordia was surrounded by several bracts. At stage 3, the spikelet meristems first differentiates into a pair of glumes in a 1/2 alternate arrangement (Figure 2D). For grasses such as wheat and rice, several florets are produced after differentiation of glumes. The first glume of each floret is called the lemma, and the other is the palea. In stage 3, floral initiation begins (Figure 2E–G). The meristem producing a floret is called the FM. At stage 4, the palea primordium, stamen primordial, and pistil primordium were detectable. However, there is no petals homologous organ in the grass family. Then the six stamen primordia are formed in the whorl (Figure 2H,I). During carpel primordia formation, stamens differentiate into anthers and filaments (Figure 1F–H,J,K). Finally, the carpel primordium differentiates into two stigmas (Figure 1E). The meristem without indeterminacy is then transformed to ovule primordium where megagametogenesis takes place (Figure 1F). At stage 5, the most advanced floral primordium transforms into a complete flower (Figure 1I and Figure 2L). The complete floret of *D. latiflorus* is composed of a lemma, a palea, six stamens and a pistil with feathery stigma (Figure 1K,L). The inflorescence includes a compound spike composed of several spikelets. Each spikelet (Figure 1J) contains 3 to 10 florets from which a single ﬂower was initiated in the axil of each bract of the inﬂorescence (Figure 1I).

After the previous flowering on the poles of the bamboo, inﬂorescence initiation in *D. latiflorus* started from March to June and mid-October to November at the leaf axils of the growing shoot in the same clumps. Floral primordia are minute and initiated successively so that some ﬂoral buds in an inﬂorescence are at a given developmental stage. Here, we applied a novel sampling approach, collecting a large number of flowering and vegetative branches on the poles of the flowering bamboo and choosing floral buds less than 5 mm in length. The buds in the same category display the synchronized development. This reliability of this floral induction system was confirmed by morphogenesis study using paraffin sectioniong and SEM. The tip of vegetative bud developed into the spikelet primordium. Next, the first and second glume primordium began to grow. At this time, the length of floral bud is 0–2 mm. The width of the growth tip can distinguish vegetative and early floral buds, as the growth tip of early floral buds is wider than the growth tip of vegetative buds. The subsequent development of ﬂoral primordial first gave rise to the initiation of the palea primordia when the floral buds are 2–3 mm. The palea originates from two distinct protuberances in its early development, consistent with the hypothesis that the palea originates from a double origin (Payer 1857). When the floral buds achieve approximately 3 mm in length, the central part of the floret primordium gave rise to the stamens primordia and pistil primordium. Floral buds continued to develop and formed the obvious stamens and carpel. At approximately 3 mm in length, floral buds have begun to develop the organs such as pistil and stamens. At the length of 3–4 mm, the pistil and stamen developed further.

We have chronicled in detail the developmental course from vegetative to reproductive growth in *D.* *latiflorus*. The developmental process of the spikelet and floret are shown in Table 1. Together this strongly suggests that the developmental process of spikelets first commenced with the growth of lemma and palea, after which the stamen and carpel developed. In the present study, different lengths of floral buds represent different developmental stages. The description of the development of inflorescence and spikelet is given in detail and the developmental course of each organ is categorized in the particular stage. This classification method based on morphological characteristics is thus standardized and reproducible, and will provide an excellent reference for studying the mechanism of floral transition and floral development in bamboo.

**Table 1:** Staging of the development of floral buds in *D. latiflorus.*

Stage	Length of Floral Buds	Events
2	0–2 mm	Spikelet primordium/the first glume primordium
3	2–3 mm	Primordium of the first floret/the second glume/the second floret
4	3–4 mm	Stamen primordia and pistil primordium
5	4–5 mm	Stamen primordia and pistil primordium continuing to develop into rapid increase in size

### 2.2. Isolation and Analysis of the Ten Candidate Genes

Ten candidate genes were isolated and analyzed in the present study. The putative ten candidate gene sequences (*AP1*, *TFL1*, *RFL*, *FT*, *ID1*, *FCA*, *MADS1*, *MADS2*, *SPL9*, and *EMF2*) were given the names *DlAP1*, *DlTFL1*, *DlRFL*, *DlFT*, *DlID1*, *DlFCA*, *DlMADS1*, *DlMADS2*, *DlSPL9*, and *DlEMF2*, respectively. Genes were amplified by PCR using cDNA template from *D.* *latiflorus* and specific primers (Table S1), and then directly sequenced. The nucleotide sequences of the ten genes were displayed in Table S2. By comparing with other homologous genes in the GenBank database, the identities of these sequences were confirmed. All sequences showed the same typical characteristics with the homologs in the grass family. Similarities with other orthologs are displayed in Table 2.

**Table 2:** Isolation and analysis of the ten candidate genes. PEBP, phosphatidylethanolamine-binding protein.

Gene Name	Length (bp)	Pathways	Domain	Similarity with Ortholog in Poaceae
*DlAP1*	228	Floral meristem identity genes	MADS-box	*MADS 14* (*Oryza sativa*) (99%)
*DlTFL1*	249	Floral meristem identity genes	Phosphatidylethanolamine-binding protein	*TFL1* (*Bambusa oldhamii*) (98%)
*DlRFL*	137	Floral meristem identity genes	FLO/LFY protein	*RFL* (*Oryza sativa*) (97%)
*DlFT*	150	Photoperiod pathway	PEBP	*FT* (*Triticum aestivum*) (98%)
*DlID1*	74	Photoperiod pathway	C_2_H_2_	*Ehd2* (*Oryza sativa Japonica group*) (100%)
*DlFCA*	112	Autonomous pathway	FCA-like protein	*FCA* (*Triticum aestivum*) (84.4%)
*DlMADS1*	92	Other flower gene	MADS-box	*MADS* (*Phyllostachys praecox*) (100%)
*DlMADS2*	130	Other flower gene	MADS-box	*MADS* (*Zea mays*) (80%)
*DlSPL9*	86	Other flower gene	SBP domain transcription factor	*SPL9* (Setaria italic) (86%)
*DlEMF2*	200	Other flower gene	VEFS-box of polycomb protein	*EMF2* (*Setaria italic*) (98%)

**Figure 3:**
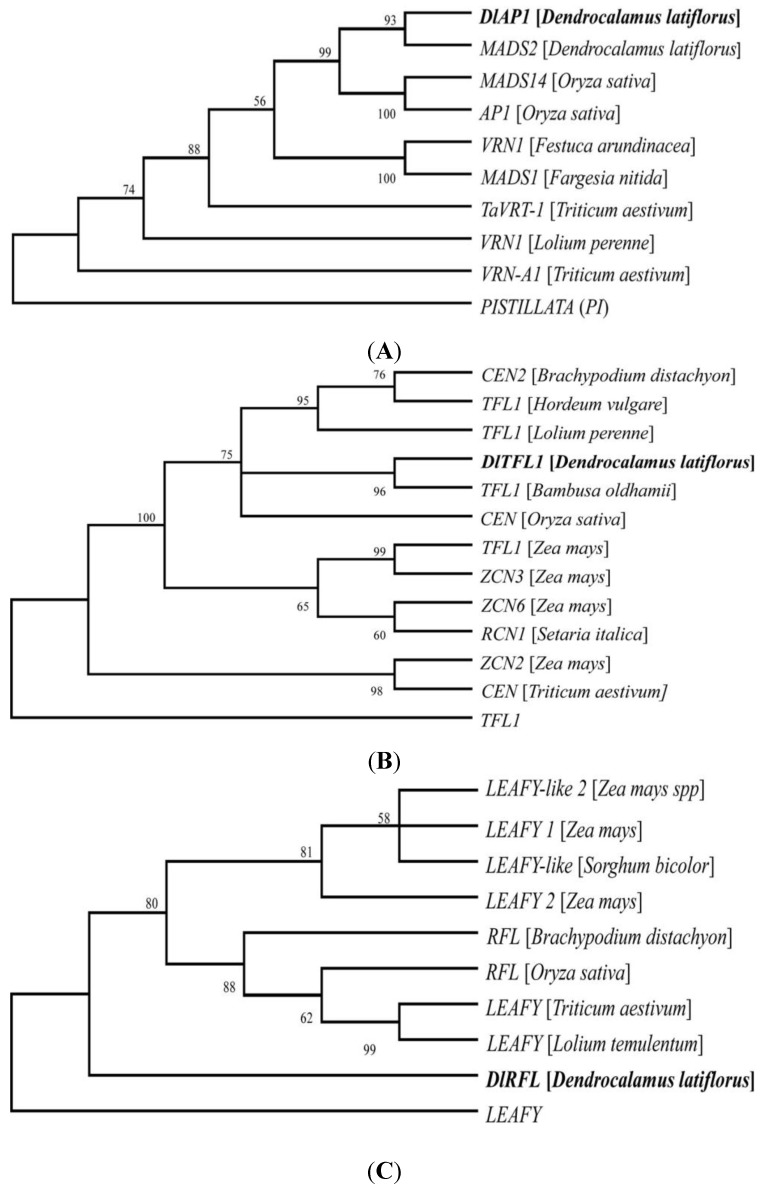
Unrooted phylogenetic trees of homologous genes in *D. latiflorus* and other grass species using NJ-method. (**A**) *AP1*; (**B**) *TFL1*; (**C**) *RFL*. *Arabidopsis* *PISTILLATA* (*PI*), *TFL1* and *LEAFY* were used as out groups. Sequences isolated in this study are marked in bold. The statistics on the branches represented bootstrap values.

*DlAP1* showed typical characteristics of the *AP1* homolog from other species, including the MADS-box domain. Conceptual translation of the ORFs of the *DlTFL1* cDNA sequence revealed a pfam 01161 domain, the phosphatidylethanolamine-binding protein (PEBP). It indicated the highest similarity to the gene *TFL1* from *B. oldhamii* (98%). *DlRFL* displayed the Pfam 1698 FLO/LFY protein domain. *DlFT* contained typical characteristics of PEBP superfamily domains. PEBPs are small globular proteins (~21 kDa) that contain an anion-binding pocket composed of highly conserved residues [27]. BLASTX searches against GenBank revealed that the deduced amino acid sequence of *DlID1* was most similar to *Ehd2*, which is a rice ortholog, and *DlFCA* was most similar to the flowering time control protein FCA in *Triticum aestivum*, with 84.4% identity. *DlMADS1* and *DlMADS2* contained MADS-box domains. *DlEMF* contained Pfam 09733, the VEFS-box of polycomb protein. The VEFS-box is located in the *C*-terminal region of polycomb proteins, and is characterized by an acidic cluster and a tryptophan/methionine-rich sequence, the acidic-W/M domain. *DlSPL9* was most similar to squamosa promoter-binding protein-like (SBP domain) transcription factor family protein. From phylogenetic tree (Figure 3A) and multiple sequence protein alignment (Figure S1) analyses, *DlAP1* showed the highest similarity to the *AP1-*like protein *MADS14* from *Oryza sativa* (99%). *MADS14* is the homolog of *AP1* in *Arabidopsis* [28,29]. Using the isolated *DlTFL1* sequence fragment and other *TFL1-*like sequences in the GenBank database, we constructed the phylogenetic tree (Figure 3B). Phylogenetic analysis revealed that the sequences of *DlTFL1* are closer to *Bambusa oldhamii TFL1*. It indicated the highest similarity to the gene *TFL1* from *B. oldhamii* (98%). The phylogenetic tree constructed using the isolated *DlRFL* nucleotide sequence and various *RFL*-like sequences downloaded from GenBank clustered the grass family Poaceae together (Figure 3C). Sequence analysis of the ten genes strongly suggests that these genes are homologs of genes in Poaceae, with high similarity. The combined data obtained by BLAST searches against the GenBank database, multiple sequence comparison and phylogenetic analysis suggest that the FM identity gene fragments in *D. latiflorus* are most likely homologs of *AP1*, *TFL1*, and *RFL* from *Oryza sativa* and *Bambusa oldhamii*.

The functions of these genes have been verified in monocot and dicot species for early flowering [8,18,19,21,23,25]. They played important roles in the floral transition and floral development. The expression level of these genes displayed certain regularity during vegetative to reproductive growth. Zhang *et al.* [30] identified 290 genes that were associated with the floral transition and 47 related to flower development in *D.* *latiflorus*. According to Zhang’s paper, other candidate genes in the present study were assigned to different pathways. We selected representative genes that were involved in the floral transition from different metabolic pathways to study. *AP1*, *TFL1*, and *RFL* homologs in *D. latifloru*s were classified into the FM identity genes. *DlID1* was assigned to the photoperiod pathway. *DlFCA* plays a vital role in the autonomous pathway. Other genes, including *DlMADS1*, *DlMADS2*, *DlSPL9*, and *DlEMF2*, were included as other flowering genes (Table 2). According to Zhang’s study, *FT* has not been discovered by transcriptome sequencing. The *DlFT* gene was detected by RT-PCR in the vegetative and floral buds in the present study, and *DlFT* was assigned to the photoperiod pathway [19].

### 2.3. Quantitative Expression Patterns of the Ten Candidate Genes from Floral Transition to Early Floral Development

**Figure 4:**
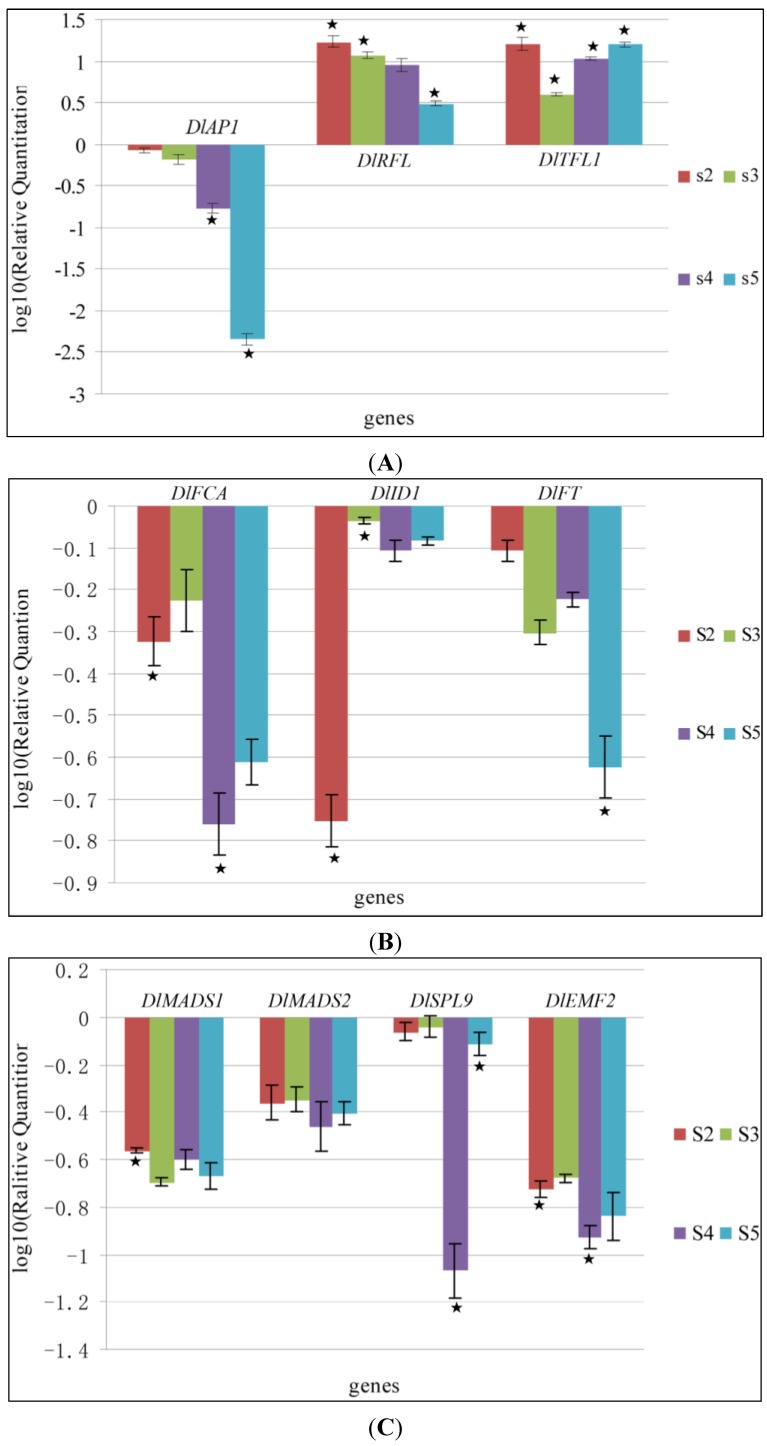
Ten candidate gene expression patterns in different stages in *Dendrocalamus latiflorus* (mean ± standard error; *n* ≥ 3). Stage 2 (S2), initiation of inflorescence; Stage 3 (S3), initiation of floral primordia; Stage 4 (S4), initiation of the primordia of stamen and pistil; Stage 5 (S5), the advanced floral primordium into a complete flower. (**A**) *DlAP1*, *DlRFL* and *DlTFL1*; (**B**) *DlFCA*, *DlID1*, and *DlFT*; (**C**) *DlMADS1*, *DlMADS2*, *DlSPL9* and *DlEMF2*. Expressions of genes in stages 2, 3, 4, and 5 were compared to stage 1. The data presented are the means of three technical replicates. The relative mRNA levels were determined using qPCR and normalized using β-actin as internal control. The black star (★) indicates significant difference compared with the previous stage by two-sample *t*-test (*p* value < 0.05).

Plant development progresses through distinct phases (vegetative growth, a reproductive phase, and senescence), and the transitions between these phases are controlled by distinct genes [25]. We next analyzed the gene expression levels from the floral transition and early development. Detailed expression profiles of then ten candidate genes from the initiation of the vegetative meristem (stage 1) to early floral development (stage 2–5) were analyzed by real-time PCR during a complete growth cycle (Figure S2). Melting curve analysis revealed a single peak for each gene in this study (Figure S3A–K), indicating that only one PCR product specific to each target or reference gene was produced in the qPCR reactions. The relative expression of vegetative buds (stage 1) was zero. Expression levels of these genes in stage 2–5 were compared to stage 1. Expression patterns of the ten genes in *D.* *latiflorus* were grouped into two categories (Figure 4): negative expression (*DlAP1*, *DlFCA*, *DlID1*, *DlFT*, *DlMADS1*, *DlMADS2*, *DlSPL9*, and *DlEMF2*) and positive expression (*DlRFL* and *DlTFL1*). Expression patterns corresponded to morphological change; distinct expression profiles of putative floral initiation homologues corresponded to the developmental stages defined by bud length.

The expression patterns of the genes varied between development stages. Additionally, using the method of two sample *t*-test [31], the *p*-values were displayed in Table S3. Most of these genes that including *DlRFL*, *DlTFL1* (Figure 4A), *DlFCA*, *DlID1* (Figure 4B), *DlMADSA1* and *DlEMF2* (Figure 4C) showed significant changes from the vegetative to reproductive growth (stage 1 to 2). Of these genes, *DlFCA*, *DlID1* (Figure 4B), *DlMADSA1* and *DlEMF2* (Figure 4C) showed lower expression in stage 2. The *DlAP1* and *DlRFL* transcripts (Figure 4A) displayed continually decreasing expression during inflorescence and floral developmental stage (stage 2–5). The transition from inflorescence to FM corresponds to stage 2 to 3, and is the first step in which an inflorescence meristem gives rise to a FM during flower development [32]. The expression level of *DlID1* (Figure 4B) was significantly increased from stage 2 to 3, while *DlRFL* and *DlTFL1* (Figure 4A) transcripts decreased during this transition. The other genes did not show any apparent changes in expression from stage 2 to 3. During floral development, expression of most of the genes (*DlAP1*, *DlTFL1*, *DlFCA*, *DlSPL9*, and *DlEMF2*) exhibited a fast growth curve during the initiation of floral primordia to the commencement of the stamens and pistil, as determined by tracking the length of the floral buds (between 2–3 and 3–4 mm). Expression of *DlAP1* (Figure 4A), *DlFCA* (Figure 4B), *DlSPL9* and *DlEMF2* (Figure 4C) showed a downward trend compared with stage 3, while *DlTFL1* (Figure 4A) showed a rapid increase at this stage. As the floret further developed (stage 4 to 5), *DlAP1*, *DlRFL* (Figure 4A), and *DlFT* (Figure 4B) transcripts displayed downward trends, while the expression of *DlTFL1* (Figure 4B) and *DlSPL9* (Figure 4C) showed a sustained upward trend. *DlTFL1* and *DlSPL9* gene expression rapidly increased after all organ initiation had completed. The expression levels of *DlMADS2* did not show any statistically significant changes during the entire developmental process.

Wang *et al.* demonstrated that *SPL9* transcript levels increase during development and are found in the shoot apical region and in young ﬂowers [33]. Fornara *et al.* [34] showed that the level of *SPL9* is low during the juvenile phase and high during the adult phase. Consistent with these studies, *DlSPL9* transcripts were expressed higher in the fully developed flower (stage 5) than during commencement of primordial stamens and pistil (stage 4). *DlEMF2* is a necessary gene that is active during the vegetative growth, while inhibited during reproductive growth [23]. These observations are consistent with the current study, in which lower expression of *DlEMF*2 was detected during the reproductive growth compared with the vegetative growth. The *ID1* gene is one of the earliest reported genes that is expressed in leaf tissues and affects the floral transition in the shoot meristem [35]. The *ID1* transcript is detected only in young leaves in maize [36]. In the present study, the *DlID1* transcript displayed a statistically significant decrease from stage 1 to 2, corresponding to development phases from vegetative to reproductive growth. The expression in the floral development also showed low expression compared to stage 1 (vegetable buds).

*AP1*, *TFL1*, and *RFL1* (in our paper, *DlRFL*, *DlTFL1*, and *DlAP1*) are involved in both FM and floral organ development. *FLORICAULA* (*FLO*) of Antirrhinum and its *Arabidopsis* counterpart *LEAFY* (*LFY*) seem to play the most important role in the establishment of floral fate. Our results (Figure 4A) imply that expressions of *DlRFL* gene at stage 2 are significantly higher than at stage 5. This is consistent with previous reports that *RFL* is expressed predominantly in very juvenile panicles but not in mature florets [37,38]. The transcript level of *TFL* has been readily detected in vegetative tissues of adult plants, such as rice, apple, or ryegrass [21,39,40]. Pillitteri *et al.* (2004) reported that *CsTFL* transcripts did not accumulate in any of the vegetative tissues in citrus, though transcripts accumulated in all organs of fully developed ﬂowers [41]. Conversely, Bradley and Ratcliffe *et al.* showed *TFL1* gene expression not only in flower development, but also vegetative phase in *Arabidopsis*. Its expression is weak during early development, however, and increases at later stages to maintain inflorescence meristem [42]. These results in *Arabidopsis* are consistent with our present study (Figure 4A). In our experiment, *DlTFL1* transcripts accumulated in fully developed flowers (stage 5). The roles of *TFL1* in different species have not been functionally determined, so the evolution of *TFL1* function is not known. The ratio of *LFY*/*TFL1* determines the meristem fate; in *Arabidopsis*, when the floral meristem produced, *LFY*/*TFL1* reached a higher ratio [41,43]. In the current study, the ratio of *LFY*/*TFL1* (Table S4) was the highest at stage 3 during the shift from inflorescence to FM (Figure 4A). *TFL1* and *FT* have opposite actions in determining the identity of the shoot meristem [44]. In our study, the expression levels of *DlTFL1* and *DlFT* displayed opposite trends. The *FT* gene was taken as a florigene, and is expressed in the vascular tissues of leaves [45,46,47]. FT protein interacts with the FD bZIP transcription factor and is only expressed in the SAM [48,49]. In our study, *FT* gene was detected in the reproductive phase. However, the expression level of *DlFT* was reduced compared to the vegetative phase. It is likely that FT protein moves from SAM to the FM in a low amount in bamboo.

*AP1* plays a critical role in the transition from vegetative to reproductive phase [50,51]. Although the gene of *AP1* in rice and maize have been cloned [52,53,54], there has been no report of a detailed analysis of either their expression or their fuction. In wheat, the expression levels of *TaVRT-1* [55] and *WAP1* [56] are positively associated with the vernalization response, and in the transition from vegetative to reproductive phase. In response to vernalization, *AP1* homologues in *Lolium perenne* are up-regulated in the shoot apex [57]. This consisted with our result the expression of *DlAP1* showed a decrease during the induction of the reproductive phase (stage 1 to 2). At the stage 1, the vegetative buds displayed the transitional shoot meristem (TSAM). This would suggest that the fate of TSAM will assume a floral fate if the inductive signal is potent enough. Maybe it is the cause of the gene expression level of stage 1 higher than the stage 2. An extension of this hypothesis would predict that *DlAP1* would be more efficient in the floral transition in bamboo species and remains to be tested in future experiments. The expression of *DlAP1* continually decreased during floral development (stage 2 to 5), and *DlAP1* mRNA levels presented a sustained downward trend from the vegetative to reproductive process (Figure 4A). The *AP1* gene, which encodes a transcription factor with a putative MADS-box domain, is normally expressed in young flower primordia, but is absent from the inflorescence meristem in *Arabidopsis* [58]. In our study, the *DlAP1* transcript is expressed both in the stages of inflorescence meristem (stage 2) and floral primordia (stage 3). However, the gene expressions between the two stages did not show a significant difference (Figure 4A). Differences in the flowering gene expression patterns indicate that *Arabidopsis* that based on flowering model is not completely applicable to explain the molecular events leading to floral transition in bamboo. We speculated that possibility is the difference of many features of floral and inflorescence development between monocot and dicot species. *DlAP1* demonstrated a sustained downward trend and could serve as a good molecular marker during floral transition in *D. latiflorus*. Together, this suggests that *DlAP1* is a direct link between the process of ﬂoral induction and the regional events associated with the initiation of individual ﬂowers.

## 3. Experimental Section

### 3.1. Experimental Materials

**Figure 5:**
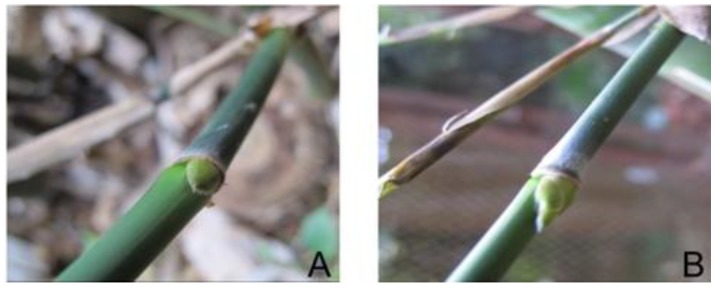
Vegetative buds and ﬂoral buds (<5 mm): (**A**) vegetative bud; (**B**) floral bud.

In October 2009, ten clumps of *D. latiflorus* in blossom were transplanted from Longsuo village, Pengpu town, Mile county of Yunnan Province in the southwest of China to the East Garden of Kunming Institute of Botany, Chinese Academy of Sciences. All transplanted bamboos were watered twice a week. As the change from vegetative to reproductive phase takes place, the crown and lateral buds change to floral buds. Young ﬂoral buds are hidden by older, more mature ﬂowers, representing an additional challenge for dissection. We chose some non-flowering branches with vegetative buds (Figure 5A) and some flowering branches with floral buds (length ≤ 5 mm) (Figure 5B). The buds were taken from the same ramet in one clump. These floral buds were divided into different developmental stages by length (0–2; 2–3; 3–4; 4–5 mm) under anatomical lens. Samples were immediately frozen in liquid nitrogen and stored at −80 °C until RNA extraction. Part of the buds stored in FAA (formalin:glacial acetic:50% ethanol = 1:1:18) were used for morphology analysis and the remaining buds were used for molecular analyses.

### 3.2. Paraffin Sectioning

Vegetative and Inflorescence buds at various lengths were fixed in FAA and dehydrated in a graded ethanol series. The samples were then embedded in Paraplast by LEICA EG 1160 (Leica Micosystems, Wetzlar, Germany). The paraplast was cut into 5–10 μm sections on a Leica RM2135 microtome and mounted on glass slides. The sections were then deparaffinized with xylene, stained with fast green, and analyzed using a Leica DFC 295 camera (Vashaw Scientific Inc., Wetzlar, Germany) attached to a Leica DM 1000.

### 3.3. Scanning Electron Microscopy (SEM)

The samples were processed for SEM as previously described [59]. Samples were dissected in 95% (*v*/*v*) alcohol and dehydrated using a series of ISO-amyl acetate, followed by critical point drying. The samples were coated in gold-palladium, and analyzed by a Hitachi S-4800 scanning electron microscope (Hitachi High-Technologies Corp., Tokyo, Japan).

### 3.4. Primary Screening for Candidate Genes

Total RNA was extracted from vegetative buds and various lengths of inﬂorescence buds using a modiﬁed RNAiso Plus (TaKaRa, Dalian, China). Frozen sample (50–100 mg) was quickly ground in a mortar and transferred to a 1.5 mL RNAase-free centrifuge tube. Reagents were added to the tube following the RNAiso Plus protocol. The total RNA was digested with DNase (Promega, Madison, WI, USA). The quantity and quality of RNA were determined using a NanoDrop spectrophotometer (Nyxor, ND-1000, Paris, France). RNA integrity was checked by visualization on a 1.5% (*w*/*v*) agarose gel containing 2% (*v*/*v*) formaldehyde.

Total RNA (0.2–2 µg) was used for cDNA synthesis along with 1 µL of 50 µM oligo dT primer and 1 µL of 40 U M-MuLV reverse transcriptase (BioTeKe Corporation, Bejing, China). cDNA (1 µL) was used as template for RT-PCR with the following program: 94 °C for 5 min followed by 35 cycles 94 °C for 15 s; 59 °C for 15 s, and 72 °C for 15 s, with ﬁnal extension at 72 °C for 5 min. PCR products were checked on a 1.5% (*w*/*v*) agarose gel with ethidium bromide staining.

All PCR products were directly sequenced at least once in an ABI 3730 sequencer (Applied Biosystems, Foster City, CA, USA). The identiﬁcation of closely related sequences in the dataset was based on BLASTX [60].

cDNA sequences were blasted and amino acid sequences were deduced by searching against the GenBank database at the National Center for Biotechnology Information (http://www.ncbi.nlm.nih.gov/BLAST).

### 3.5. Sequence and Phylogenetic Analysis of Meristem Genes (DlAP1, DlTFL, DlRFL)

Homologous sequences of each of the three newly isolated meristem genes (*DlAP1*, *DlTFL*, *DlRFL*) fragments from *D. latiflorus* were obtained from GenBank. Each sequence was manually checked and errors corrected using BioEdit (Ibis Biosciences, Carlsbad, CA, USA) to improve the quality and reliability. Sequence alignments were conducted using Clustal X [61]. The phylogenetic trees of homologous genes were constructed employing the neighbor-joining method of MEGA3.1 [62] with 1000 bootstrap replicates. *Arabidopsis PISTILLATA* (*PI*), *TFL1* and *LEAFY* were used as outgroups of *DlAP1*, *DlTFL1* and *DlRFL* sequences, respectively. The genes used in this study are listed in Table S5.

### 3.6. Quantitative Real-Time PCR

All the primers were spaced within a range of 70–200 nucleotides. Each of the templates was diluted into the same concentration. The FastStart DNA Master SYBR^®^ Green I (Roche, Laval, QC, Canada) was used for qPCR reactions performed in the 96-well plate in a 20 μL reaction volume. A 20-fold dilution of the reverse transcription reaction was used for Real-time analysis. The procedure of real-time PCR was as follows: an initial denaturing step at 95 °C for 10 min, followed by 40 cycles of 95 °C for 15 s, 60 °C for 60 s, 72 °C for 30 s. The bamboo Pp *ACT1* [1] gene homologous to β-actin was renamed as *DlACT1.* *DlACT1* was used as a control to normalize the amount of total transcripts in each sample. All primers used in qRT-PCR are listed in Table S1.

To handle the limitations of SYBR green technology melting curve analysis was performed at a higher temperature (80–85 °C) to nullify the possibility of primer–dimer formation. Quantiﬁcation of mRNA was based on the threshold cycle (*C*_t_) value of each sample using the relative quantiﬁcation method [63]. To minimize experimental error, PCRs containing cDNAs from a series of experimental samples were generally included in a single PCR run, with genes of interest and housekeeping genes sharing the same cDNA template in each of the samples investigated. A widely used method to present relative gene expression is the comparative *C*t method also referred to as the 2^−ΔΔ*C*t^ method [64,65]. The relative quantitative method was used in the analysis of gene expression profiling and the expression quantity was calculated using the level of the gene in vegetative bud (stage 1) as a calibrator. The relative expression of vegetative buds is zero. The relative quantiﬁcation analysis was performed using 7500 system SDS software version 4.0 (Applied Biosystems). The ratio of the target gene to the reference gene (*DlACT1*) in an unknown sample was compared with the ratio of the same two genes in a sample referred to as the calibrator (*i.e.*, vegetative buds in the current experiments) [13]. The significance of gene expression was analyzed using *t*-test of R software [31].

## 4. Conclusions

The developmental stages of *D. latiflorus* buds were marked by morphological changes that were used to distinguish between vegetative and reproductive states. Gene expression profiling based on the stages of different morphological traits identified *DlAP1* as an excellent marker of flowering in this species, as its expression exhibited a continually decreasing trend during floral transition and early floral development. This is the first gene marker identified for directing flowering initiation in *D. latiflorus*. Further work is necessary to identify additional genes that may show significant expression during bamboo floral development. The system holds the promise of improving our understanding of the floral transition in bamboo, while also providing insight to advance bamboo forest protection and reproduction.

## Supplementary Files

Supplementary File 1
