# Supplementary material for: Morphology and Quantitative Monitoring of Gene Expression Patterns during Floral Induction and Early Flower Development in Dendrocalamus latiflorus
Source: Int J Mol Sci. 2014 Jul 7;15(7):12074–93. doi: 10.3390/ijms150712074 (PMC4139830; doi:10.3390/ijms150712074)

Supplementary Information

Table S1. Primers used for gene cloning and PCR analysis in the present study. The primers were designed by the software Primer Express 3.0 on the basis of the sequences conserved in the orthologs (such as *Oryza sativa*, *Triticum aestivum*, *Lolium temulentum*) of these genes.

Primer Name	Primer Sequence (5'–3')
DlAP1 F	ATCGAGAACAAGATCAACCGGCAGG
DlAP1 R	CCTTCAGTTTCCTATATTCGTGGCA
DlTFL1 F	TGGTTATGACGGACCCAGATG
DlTFL1 R	TACCGATGTTCGGCTTTGG
DlRFL F	AGCCCAAGATGCGGCACTA
DlRFL R	AGACGGCGTCGATGTCGAA
DlID1 F	GGAAGGCGCACGTCAAGAAC
DlID1 R	TCAGCTAGGGCATCGCAGAAG
DlFT F	GATGTGCTACGAGAGCCCAAGG
DlFT R	CGCTGGCAGTTGAAGTAGACGG
DlMADS1 F	GGAACGGGCTGCTCAAGAA
DlMADS1 R	CCATATTACTTGCTTGGCTGGAGTA
DlMADS2 F	CATGTATGGACAAAATTCTTGAACG
DlMADS2 R	GGTGCTTTTGACATTTCTGTATCG
PpACT F	GTGTTTGGATTGGAGGGTCCATCCT
PpACT R	GTAGCTTACATGGCAAGGACTTG
DlEMF2 F	TACTACAGTTTGGGAAGACAAGGAA
DlEMF2 R	CCGAACAAATGAATTCCACATATGC
DlSPL9 F	ACTGTGGTAGTTGCTGCTGTAT
DlSPL9 R	GTGGAGTCGCTAGACAAGTACC
DlFCA F	TGCCTGCTGTGAAGATTGATAAC
DlFCA R	CAAGTTGTTCCTGGCCAACA

Table S2. The nucleotide sequences of the candidate genes that isolated.

Name	Sequence (5'–3')
DlAP1	CGCGGTCGGAGCTGCTCAGAGGCGCATGAGATCTCCGTCCTCTGCGACGCCGAGG<br>TCGGCCTTATCATCTTCTCCACCAAGGGCAAGCTCTACGAGTACGCCACCGACTCA<br>TGTATGGACAAAATTCTTGAACGGTACGAGCGTTACTCCTATGCAGAAAAGGTTCT<br>TATTTCAGCCGAATCTGAAACTCAGGGCAACTGGTGCCACGAATATAGGAAAACT<br>GAAGGA
DlTFL1	AGCTCTACCCATCAGCAGTTGAATCTAAACCAAGAGTAGAGGTCCAAGGGGGTG<br>ACTTGCGGTCTTCCTTCACATTGGTTATGACGGACCCAGATGTGCCAGGACCAA<br>GTGATCCATACCTAAGGGAGCACCTTCACTGGATTGTCTCTGATATACCTGGGAC<br>AACAGATGCTTCTTTTGGACGAGAGGTCATAAGCTACGAGAGCCCAAAGCCGAA<br>CATCGGTATCCACAGGCTTCATTTTTAAAAAC
DlRFL	TGGCAGAGGAGGCGTCCAACGCGCATGCGGCGGGCGTACAAGGCGCGCGGCGA<br>GAACGTCGGCGCGTGGCGGCAGGCGTGCTACGCGCCGCTCGTCGACATCTCCGC<br>GCGCCACGGCTTCGACATCGACGCCGTCTA
DlEMF2	CTTCCGTTCTCACAGGGCACAGCCAATGGCATTGGAACAAGTTTTTTCAGATCGT<br>GATAGTGAAGACGAAGTTGATGATGATATCGCCGACTTTGAAGATAGAAGGATG<br>CTTGATGATTTTGTTGATGTTACAAAAGATGAGAAGCTTATTATGCATATGTGGA<br>ATTCATTTGTTCGGAAACAAAGGGTGCTAGCCGATG
DlFT	ATGTGCTACGAGAGCCCAAGGCCAACCATGGGGATCCACCGCTTCGTGTTCGTG<br>CTGTTCCAGCAGCTGGGCCGGCAGACGGTGTACGCCCCCGGTTGGCGCCAGAAC<br>TTCAACACCAGGACTCGCCGAGCTCGACATGCTGACCCTACG
DlID1	TGGAAGGCGCACGTCAAGAACTGCGGCACGCGCGAGTACCGTTGCGACTGCGG<br>CATCCTCTCTCCAGAAGACAG
DlFCA	GCAGCTGTTGGAGTAGCACTGTGGTGCTTGTGACGGCATCTGCTGCATTGGCTGT<br>TGAAGCATTTGCGGTGCAACATTGGAGGGAAGTTGCTGTTGGCCAGGAACAACT<br>TGA
DlSPL9	CTGGTTGCATCACTTATAACATTCACCCACAGAGAGTAGGGGTGCTTGCAGCTC<br>CAGTGAAGAGGTACTTGTCTAGCGACTCCACA
DlMADS1	TGACTCGCGCTCTGCGACGCCGAGGTCGCGCTCATCGTCTTCTCCACCAAGGGC<br>AAGCTCTACGAGTACTCCAGCCAAGCAAGTAATATGGA
DlMADS2	GGCAACTGGTGTCACGAATATAGGAAACTGAAGGCGAAGGTTGAGACGATACA<br>GAAATGTCAAAAGCACCATG

Table S3. The p-value of the genes between two stages by the two-sample *t*-test. S1: the vegetative phase; S2: initiation of inflorescence; S3: initiation of floral primordia; S4: initiation of the primordia of stamen and pistil; S5: the advanced floral primordium into a complete flower. The black star (★) indicates significant difference compared with the previous stage (p value < 0.05).

Genes	S2/S1	S3/S2	S4/S3	S5/S4
DlAP1	0.7149	0.0659	0.0003 ★	0.0000 ★
DlRFL	0.0000 ★	0.022 ★	0.1028	0.0468 ★
DlTFL1	0.0392 ★	0.0001 ★	0.0000 ★	0.0014 ★
DlFCA	0.0038 ★	0.1459	0.0009 ★	0.6727
DlID1	0.0005 ★	0.0000 ★	0.6442	0.8834
DlFT	0.6333	0.4300	0.6990	0.0211 ★
DlMADS1	0.0082 ★	0.3132	0.3571	0.1343
DlMADS2	0.0750	0.8168	0.1660	0.448
DlSPL9	0.2707	0.5757	0.0001 ★	0.0002 ★
DlEMF2	0.0105 ★	0.0931	0.0013 ★	0.2520

Table S4. The ratio of RFL/TFL1. S2: initiation of inflorescence; S3: initiation of floral primordia; S4: initiation of the primordia of stamen and pistil; S5: the advanced floral primordium into a complete flower.

Expression Quantity	S2	S3	S4	S5
FRL	1.234	1.061	0.95	0.487
TFL1	1.206	0.595	1.03	1.2
RFL/TFL1	1.02	1.78	0.92	0.41

Table S5. *AP1*, *TFL, RFL* homologous genes used for sequence comparison and phylogenetic analysis in this study.

Gene	Taxon	Accession No.	Gene	Taxon	Accession No.
PISTILLATA (PI)	*Arabidopsis thaliana*	NM_122031.3	*TFL1*	*Zea mays*	DQ925416.1
VRN1	*Festuca arundinacea*	FJ793194.1	*ZCN3*	*Zea mays*	NM_001112771.1
VRN-A1	*Triticum aestivum cultivar*	JF965397.1	*TFL1*	*Arabidopsis thaliana*	NM_125597.1
MADS1	*Lolium temulentum*	AF035378.1	*RFL*	*Brachypodium distachyon*	XM_003580387.1
MADS14	*Oryza sativa*	AF058697.1	*RFL*	*Oryza sativa*	AB005620.1
AP1	*Oryza sativa*	AB041020.1	*LEAFY*	*Triticum aestivum*	AB231889.1
MADS2	*Dendrocalamus latiflorus*	AY395715.1	*LEAFY2*	*Zea mays*	AY789030.1
VRN1	*Lolium perenne*	GQ227990.1	*LEAFY2*	*Zea mays* spp.	AY789047.1
TaVRT-1	*Triticum aestivum*	AY280870.1	*LEAFY*	*Lolium temulentum*	AF321273.1
TFL1	*Bambusa oldhamii*	HM641253.1	*LEAFY-like*	*Sorghum bicolor*	XM_002446991.1
CEN2	*Brachypodium distachyon*	XM_003578853.1	*LEAFY1*	*Zea mays*	NM_001111731.1
CEN	*Oryza sativa*	AF159882.1	*LEAFY*	*Arabidopsis thaliana*	NM_125579.1
TFL1	*Hordeum vulgare*	AB447465.1	*CEN*	*Triticum aestivum*	AJ577366.1
TFL1	*Lolium perenne*	AF316419.1	*RCN1*	*Setaria italica*	DQ317428.1
ZCN6	*Zea mays*	NM_001112774.1	*ZCN2*	*Zea mays*	NM_001112770.1

Figure S1. Alignment of predicted amino acid sequences of *DlAP1*, *DlTFL1*, *DlRFL*. The figure shows the align result of deduced amino acid sequences of the floral floral meristem indentity genes *DlAP1*, *DlTFL1*, *DlRFL* and homologs in the grass family. Identical amino acids are indicated by dots. Dl: *Dendrocalamus latiflorus*; Os: *Oryza sativa*; Fa: *Festuca arundinacea*; Fn: *Fargesia nitida*; Ta: *Triticum aestivum*; Lp: *Lolium perenne*; Zms: *Zea mays spp*; Zm: *Zea mays*; Sb: *Sorghum bicolor*; Bd: *Brachypodium distachyon*; Lt: *Lolium temulentum*; Hv: *Hordeum vulgare*; Bo: *Bambusa oldhamii*; Si: *Setaria italic*; At: *Arabidopsis thaliana*. (**A**) DlAP1; (**B**) DlTFL1; (**C**) DlRFL.

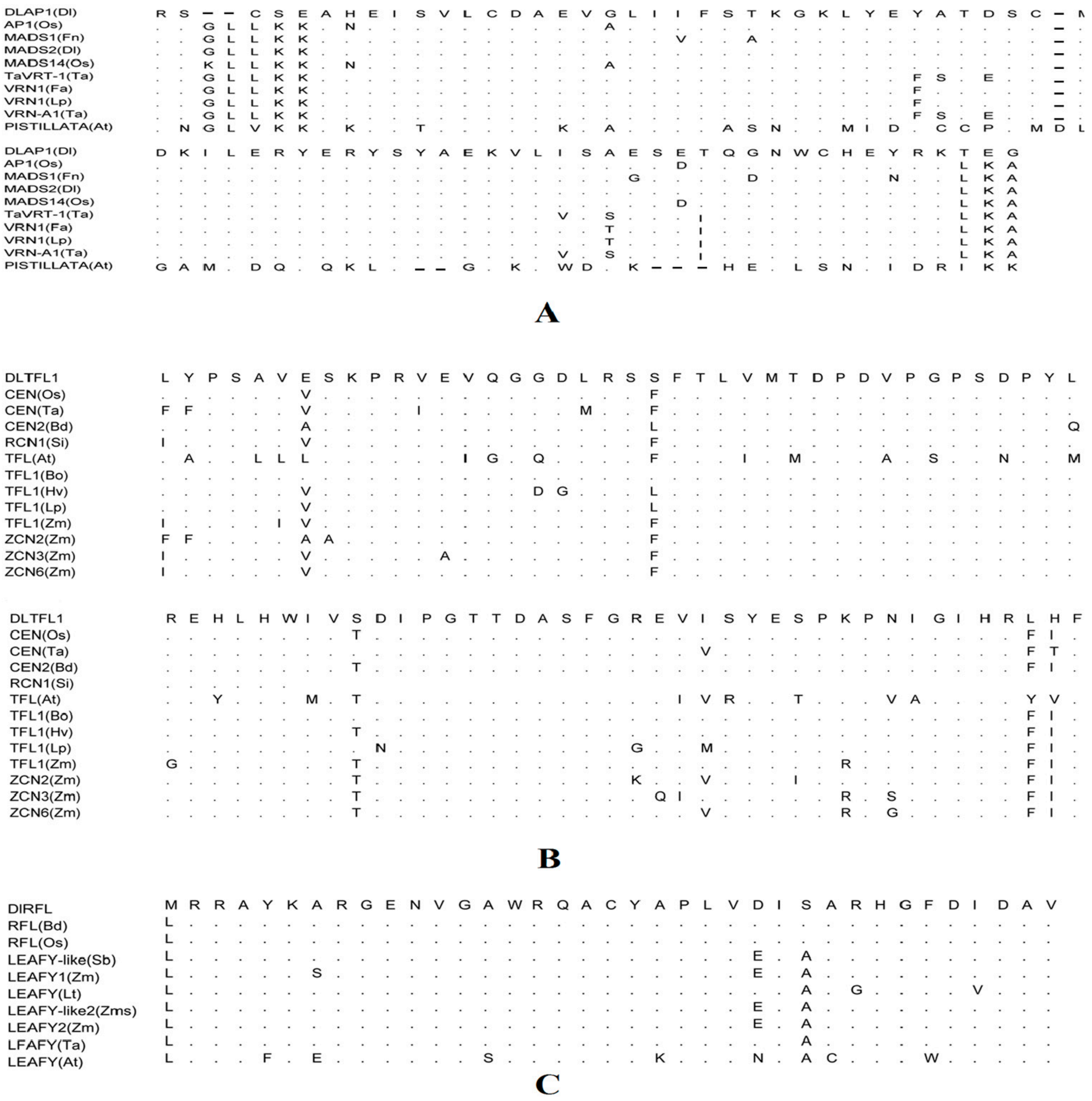

Figure S2. The electrophoresis results of RNA and Real-time PCR products. S1: vegetative buds; S2: initiation of inflorescence; S3: initiation of floral primordia; S4: initiation of the primordia of stamen and pistil; S5: the advanced floral primordium into a complete.

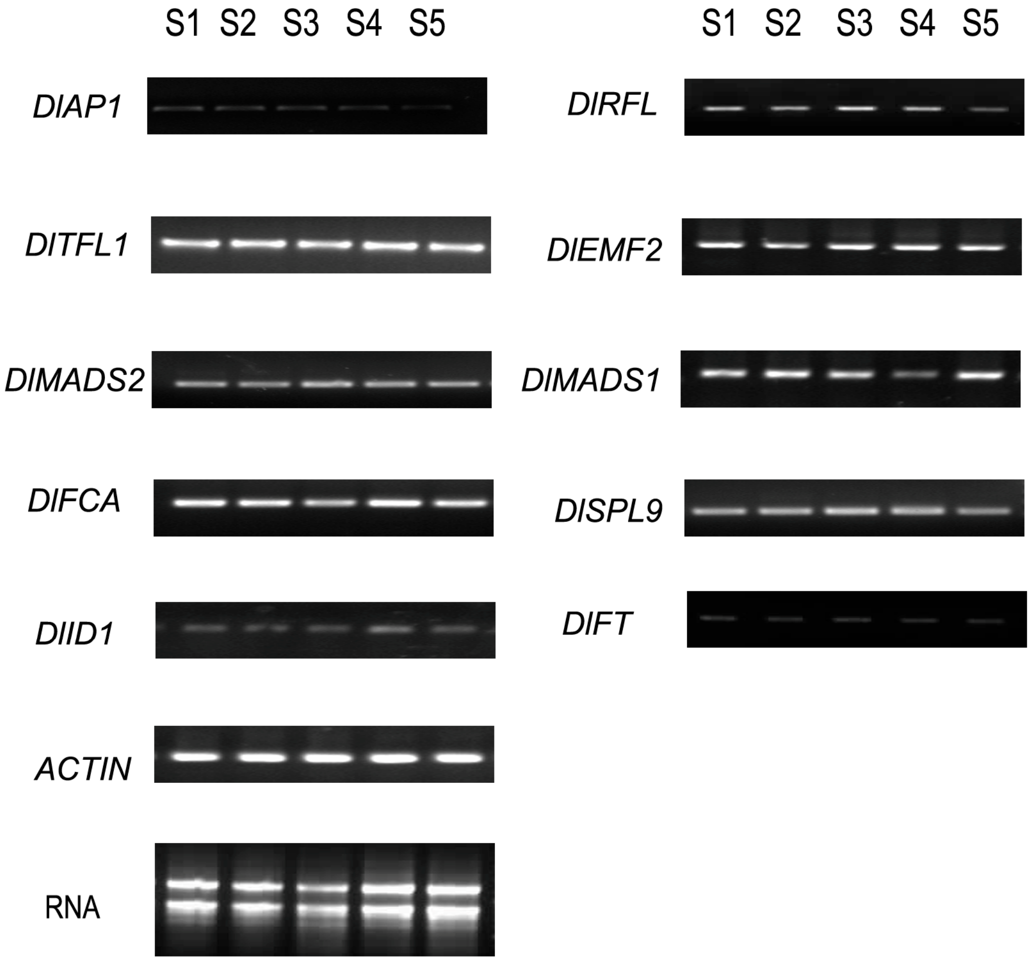

Figure S3. Melting curve analysis of ten candidate genes and *ACTIN*. (**A**) *DlAP1* (**B**) *DlMADS1*; (**C**) *DlMADS2*; (**D**) *DlEMF2*; (**E**) *DlFCA*; (**F**) *DlFT*; (**G**) *DlRFL*; (**H**) *DlSPL9*; (**I**) *DlTFL1*; (**J**) *DlID1*; (**K**) *ACTIN*.

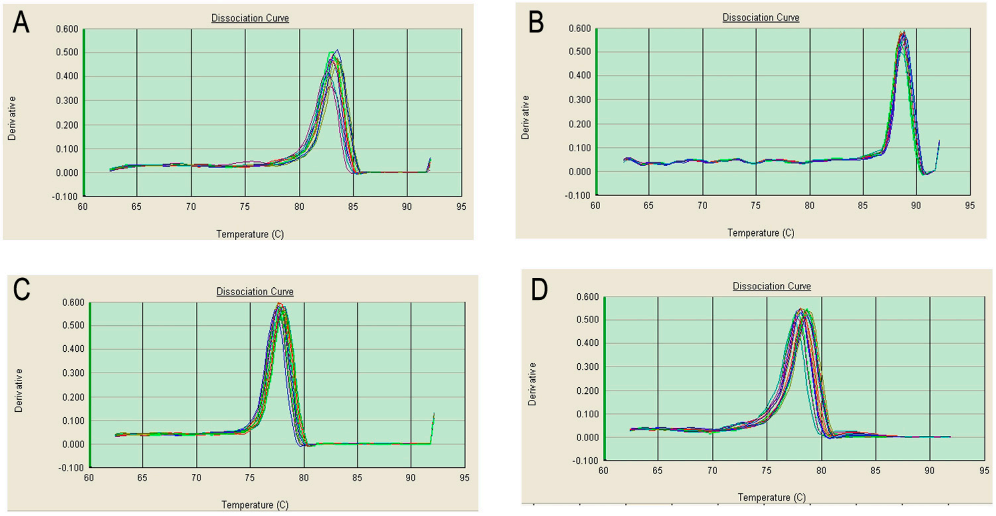

Figure S3. *Cont.*

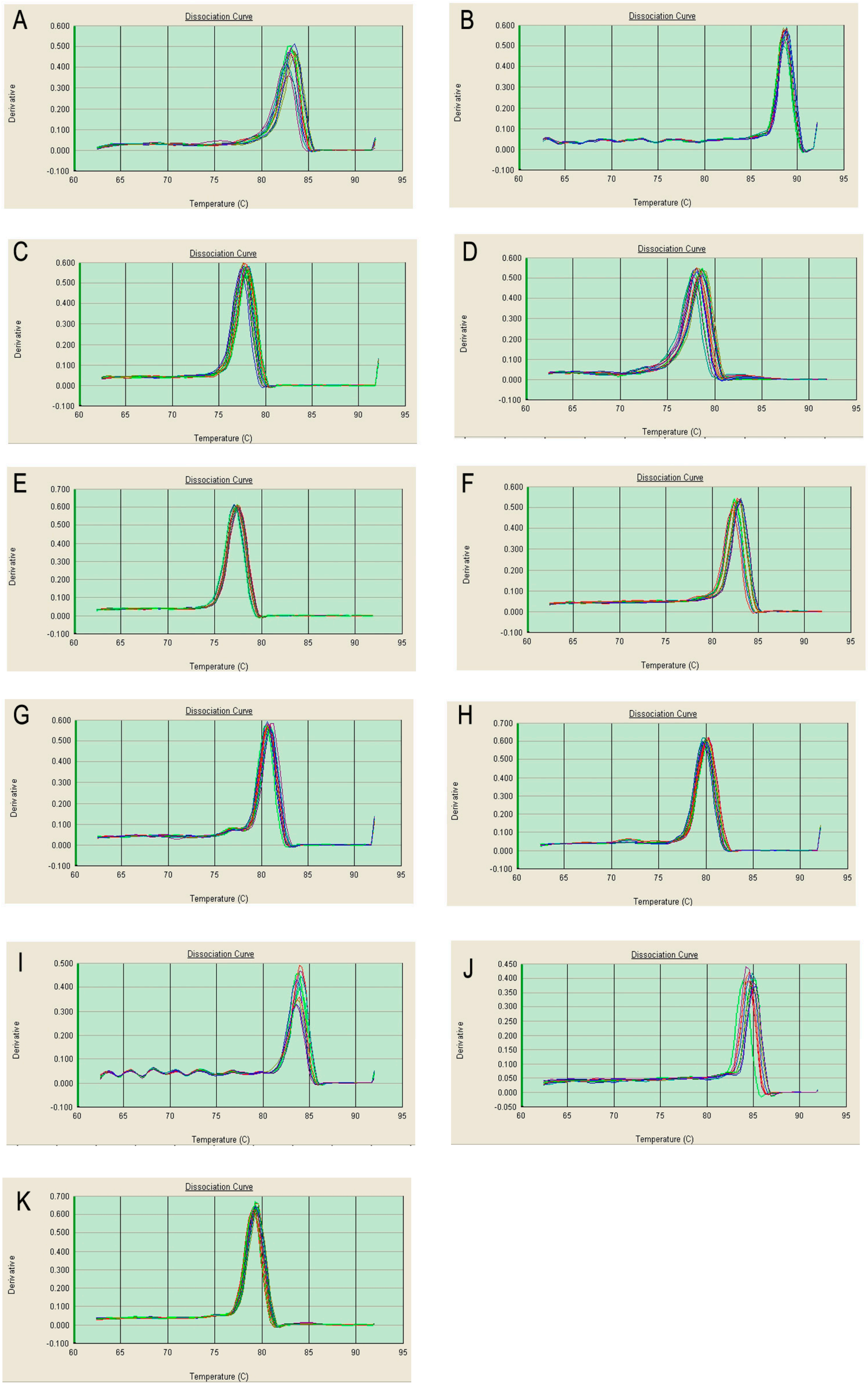

Supplement: Supplementary File 1 [file ijms-15-12074-s001.pdf]
